# Microwave-Assisted Synthesis of Water-Dispersible Humate-Coated Magnetite Nanoparticles: Relation of Coating Process Parameters to the Properties of Nanoparticles

**DOI:** 10.3390/nano10081558

**Published:** 2020-08-08

**Authors:** Egor M. Kostyukhin, Vera D. Nissenbaum, Evgeny V. Abkhalimov, Alexander L. Kustov, Boris G. Ershov, Leonid M. Kustov

**Affiliations:** 1 Laboratory of Development and Study of Polyfunctional Catalysts, N.D. Zelinsky Institute of Organic Chemistry RAS, 47 Leninsky prosp, Moscow 119991, Russia; vdn14@inbox.ru (V.D.N.); kyst@list.ru (A.L.K.); 2 Laboratory of Nanochemistry and Ecology, National University of Science and Technology MISiS, 4 Leninsky prosp., Moscow 119049, Russia; 3 Laboratory of Radiation-Induced Chemical Transformations of Materials, A.N. Frumkin Institute of Physical Chemistry and Electrochemistry RAS, 31 Leninsky Prospect, bldg. 4, Moscow 119071, Russia; abkhalimov@ipc.rssi.ru (E.V.A.); ershov@ipc.rssi.ru (B.G.E.); 4 Laboratory of Ecological Chemistry, Chemistry Department, M.V. Lomonosov Moscow State University, 1 Leninskie Gory, bldg. 3, Moscow 119991, Russia

**Keywords:** iron oxides, magnetite, humic substances, microwave synthesis, hydrophilic coating

## Abstract

Nowadays, there is a demand in the production of nontoxic multifunctional magnetic materials possessing both high colloidal stability in water solutions and high magnetization. In this work, a series of water-dispersible natural humate-polyanion coated superparamagnetic magnetite nanoparticles has been synthesized via microwave-assisted synthesis without the use of inert atmosphere. An impact of a biocompatible humate-anion as a coating agent on the structural and physical properties of nanoparticles has been established. The injection of humate-polyanion at various synthesis stages leads to differences in the physical properties of the obtained nanomaterials. Depending on the synthesis protocol, nanoparticles are characterized by improved monodispersity, smaller crystallite and grain size (up to 8.2 nm), a shift in the point of zero charge (6.4 pH), enhanced colloidal stability in model solutions, and enhanced magnetization (80 emu g^−1^).

## 1. Introduction

The use of magnetic nanoparticles has been attracting a lot of interest lately, and today such materials are used for various applications, such as toxic contaminant removal, catalysis [1,2,3,4,5,6], electronics [7,8,9], and medicine. The most widely applied examples of magnetic materials are magnetite (Fe_3_O_4_) and maghemite (γ-Fe_2_O_3_), which are now used in medicine for diagnostics and the treatment of oncological diseases, drug delivery, etc. [10,11,12,13,14].

Diverse preparation methods are used to produce magnetite/maghemite nanoparticles: thermal decomposition of organic precursors, solvothermal and hydrothermal approach, combustion synthesis, chemical precipitation and others [15,16,17]. However, the most commonly used synthesis method remains to be the classical precipitation of Fe^3+^ and Fe^2+^ from water solutions by ammonia. This approach is simple, highly efficient, and relatively fast. Nevertheless, it has several well-known disadvantages, mainly a wide size distribution of prepared nanoparticles, and as a result, differences in the physical properties due to the “size effect”.

This limitation can be overcome by using nontraditional synthesis methods, especially, a microwave approach [18,19,20,21]. It has been shown [22] that the use of microwave irradiation during the synthesis of nanomaterials allows one to obtain nanoparticles with a narrower size distribution, to control and adjust the shape and structure of nanomaterials, to reduce synthesis duration, and to enhance the yield and crystallinity of the obtained nanoparticles. Currently there is no consensus about the physical origin of specific properties of some materials obtained via microwave-assisted synthesis, nonetheless, this approach has become a routine laboratory practice.

In order to produce monodispersed nanopowders, different species added to the reaction mixture as stabilizing agents have been used. In the literature, a wide spectrum of stabilizers has been reported [23] ranging from organic to inorganic, synthetic or natural compounds.

Humic substances (HSs) include a large class of natural compounds representing 50–90% of organic substances of peat, coals, sapropel, and insentient matter of edaphic and water ecosystems. HSs are high-molecular-weight organic compounds containing a large amount of carboxylic, carbonyl, and hydroxyl functional groups, which are the result of the mineralization of organic matter of necrotic organisms [24,25].

In the literature, one can find reports on using HSs as a stabilizer for nanoparticles of metals [26,27] and oxides [28,29,30,31,32]. However, their use is not only limited to preventing aggregation of nanoparticles but it is suggested that derivatives of humic acid could play a role of a functional component, for example, in processes of water cleaning from heavy metals [33,34] and organic contaminants [35,36]. In addition, it was shown that humates have antibacterial, antiulcer, antiallergenic, and anti-inflammatory properties [37,38], inhibit cytokine production, and complement activation [24,39]. The selection of humates as stabilizing agents is often determined by their strong affinity to the surface of nanoparticles, biocompatibility (the safe daily human dose is about 1 g kg^−1^), and good colloidal stability in water–salt solutions, which prevents the aggregation and sedimentation of nanoparticles in a wide pH range [40,41,42]. Therefore, the polyanion can be a good candidate as a stabilizer in medicinal applications in vivo. There are a number of works describing the adsorption behavior of humic acid and its derivatives on iron oxides surfaces. In particular, it was concluded [43] that humic acid and its derivatives can change the surface charge of magnetite nanoparticles depending on the concentration of a stabilizer.

It is well known that the variability of the synthesis parameters (duration, temperature, pH, concentration of components) influences the final nanoparticles and their physical properties. However, the most important parameter in the preparation process is the consequence of synthetic steps. Such steps play a crucial role when we talk about the coating process of nanoparticles. However, we have not found any works aimed at studying the influence of an encapsulation protocol of magnetite/maghemite nanoparticles with the humate polyanion on the physical properties of obtained humate-coated iron oxide nanoparticles. This seems to be important, particularly in the context of hydrophilic magnetic functional materials for biomedicine, which must possess high magnetization, small size with a narrow distribution, high stability in blood, and high biocompatibility.

In view of the above-mentioned aspects, a series of one-pot syntheses of water-dispersible magnetite nanoparticles coated by a biocompatible natural polyanion, potassium humate, using conventional and microwave heating in the ambient atmosphere was accomplished in this work. It was shown that injection of humate-polyanion at various synthesis stages leads to differences in the physical properties of obtained nanomaterials. The combination of the microwave treatment and in-situ stabilization process has resulted in monodisperse water-dispersible nanopowders with high magnetization, small size with a narrow distribution, and high stability in model solutions, which can be successfully used in the biomedical field.

## 2. Materials and Methods

### 2.1. Materials

Iron(II) chloride tetrahydrate (99+%), iron(III) chloride hexahydrate (99+%) and ammonium hydroxide (25% solution in water), hydrochloric acid (37%), and sodium hydroxide (98%) were purchased in Acros Organics™, Geel, Belgium. Sodium chloride (99+%), albumin from human serum (99+%) and glucose (99.5+%) were purchased from SigmaAldrich, St. Louis, MO, USA. All chemicals were used without further purification.

Commercially available potassium humate from leonardite (a highly oxidized variety of lignites) (Powhumus^®^, Humintech GmbH, Grevenbroich, Germany) was used. Its basic properties, such as elemental and functional group analyses, ^13^C NMR spectroscopy, and size exclusion chromatography have been reported earlier elsewhere [26].

### 2.2. Synthesis of Samples

#### 2.2.1. General Procedure

All the samples were precipitated from water solutions (300 mL) of FeCl_2_·4H_2_O (4.3 g) and FeCl_3_·6H_2_O (11.6 g) by instant addition of 25 wt% NH_4_OH at an increased temperature (80 °C) [44]. Since the magnetite surface is positively charged below a point of zero charge (PZC_magnetite_ is about pH = 8) [45], while the humate polyanion is negatively charged in almost the entire pH range, and at the same time, the crystallization process of magnetite phase is most favorable in the pH range of 8–14 [46,47], the syntheses were performed in the pH range of 7.5–7.7 for successful adsorption of the polyanion onto the surface in a one-step process. When a black precipitate was obtained, a solution was aged for 30 min at the same temperature (80 °C). After that, the obtained powder was separated with a magnet and washed with distilled water. Finally, a wet black powder was placed in a baker for 12 h at 90 °C.

#### 2.2.2. Synthesis of Uncoated Magnetite Nanoparticles

Uncoated magnetite samples HS-0 and MW-HS-0 were prepared by the described above procedure. However, the synthesis of the HS-0 sample was performed by conventional heating on an oil bath with agitation, while synthesis of the MW-HS-0 sample was carried out in a microwave oven Midea AW925EHU (Midea Group, Beijiaozhen, China) equipped with an overhead quartz stirrer.

#### 2.2.3. Synthesis of Humate-Coated Magnetite Nanoparticles

The samples MW-HS-1, MW-HS-2, MW-HS-3 were prepared via microwave-assisted synthesis in a microwave oven (Midea AW925EHU) equipped with an overhead stirrer using potassium humate (110 mg) as a capping agent preliminarily dissolved in 40 mL of distilled water also by the above described procedure. A wide range of humic substances concentrations was used in the synthesis of nanoparticles, which vary from 0.1 to 3.3 g L^–1^ [28,32]. Also, there is no consensus on the ratio of NPs/HSs, which, evidently, depends on the surface area of stabilizing materials and chemical composition of HSs. Obviously, in a general case, the use of an increased amount of HSs leads to an improvement of stability, but a decrease in the saturation magnetization [32]. We proceeded from a compromise solution that allowed us to obtain stabilized nanoparticles with a high magnetization. Thus, the mass ratio of final magnetite/potassium humate equal to 40:1 has been chosen as an optimal ratio to obtain magnetite nanoparticles with a high magnetization, which form stable colloidal solutions. Potassium humate injection into the reaction mixture was performed at different preparation stages as follows: after (MW-HS-1) and before (MW-HS-2) magnetite nanoparticles precipitation, and before iron-containing salts dissolution (MW-HS-3).

### 2.3. Samples Characterization

X-ray diffraction (XRD) patterns were recorded at room temperature over the scanning range (2ϴ) of 25.0–60.0° with a step of 0.020° and scan speed of 2° min^−1^ using an ARL X’TRA (Thermo Scientific, Waltham, MA, USA) powder diffractometer equipped with a Cu anode (Kα_1,2_ irradiation, λ_1_ = 1.540562 Å, λ_2_ = 1.544390 Å). For all the samples, experimental peak profiles fitting, lattice parameters calculations, and determination of the size distribution of the crystallites were performed by the whole powder pattern modelling (WPPM) method [48,49], which is embodied in the general nonlinear least squares fitting software PM2k [50].

The samples morphology was studied using a Hitachi HT7700 (Hitachi Ltd., Tokyo, Japan) transmission electron microscope. Images were acquired in a bright-field TEM mode at a 100-kV accelerating voltage. A target-oriented approach was used for the optimization of the analytical measurements [51]. Before measurements, the samples were mounted on a 3-mm copper grid covered with a carbon film from isopropanol suspension. Size distributions were obtained from the measurement of at least 300 randomly selected particles per sample.

Infrared (IR) spectra were recorded for 32 scans with the resolution of 4 cm^−1^ using an IR-Fourier-spectrometer Nicolet iS50 (Thermo Scientific, Waltham, MA, United States) with a built-in diamond ATR.

Thermal analysis was performed by the TG-DTA method [52] using a Derivatograph-C instrument (MOM, Budapest, Hungary). The sample (100 mg) was placed in a platinum crucible and was heated in air from 20 to 600 °C at a heating rate 10 °C min^−1^.

Magnetic measurements were performed using a vibrating magnetometer described elsewhere [53]. The samples (10–20 mg) were placed between two gas-permeable quartz membranes in a flow-type quartz measuring cell of the vibrating magnetometer. The measurements were performed at an ambient temperature (300 K). The measurement accuracy did not exceed 5%.

Nanoparticle sizes as well as values of the ζ-potential in obtained colloidal solutions were determined by a dynamic light scattering (DLS) technique [54]. The measurements were performed with a Delsa Nano C particle analyzer (Beckman Coulter, Brea, CA, USA) at a wavelength of 658 nm. DLS measurements were performed in distillated water. In each experiment, 5 mg of the sample was dispersed in water (10 mL) using an ultrasonic bath. The solution pH value in the range of 2–14 was adjusted by addition of HCl and NaOH solutions. Prior to the onset of the measurements, an examined solution was thermostated at 25 °C. Colloidal stability of synthesized nanoparticles was investigated by densitometry with a spectrophotometer Cary 100 (Varian Inc., Middelburg, Netherlands) at the wavelength of 600 nm for 1 h in deionized water, normal saline solution (0.9 wt.% of NaCl), and model infusion solution consisting of albumin (100 mg L^−1^), glucose (0.5 g L^−1^), and NaCl (0.9 wt.%). Colloidal solutions were prepared by dispersion of 5 mg of nanoparticles in above mentioned solutions (10 mL) using an ultrasonic bath.

## 3. Results and Discussions

A positive influence of the use of microwave irradiation during the synthesis of nanoparticles, particularly magnetite, was demonstrated earlier [55]. Nevertheless, in this work, two synthesis approaches (conventional thermal and microwave-assisted) were performed at the same conditions for comparison. Based on electron microscopy and x-ray diffraction data (will be discussed in the corresponding sections later), we can conclude that the sample obtained via microwave-assisted synthesis exhibits a higher monodispersity. This property of nanoparticles is of great importance for their utilization in various applications, since monodisperse particles have the same relaxation time, high heating efficiency in the external electromagnetic field [56,57]. In addition, the use of microwave irradiation as a heating source allows one to reduce the synthesis time. For these reasons, the following syntheses with the stabilizing agent were performed in microwave-assisted conditions (Table 1).

### 3.1. Structural Analysis

**Figure 1:**
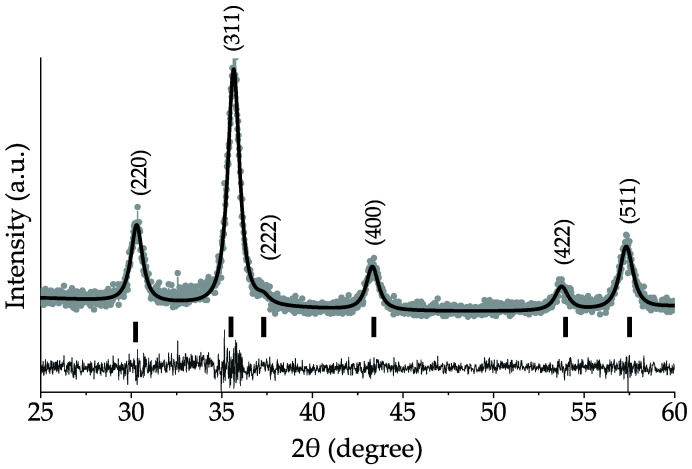
XRD pattern of the representative MW-HS-0 sample (gray dots) with WPPM fitting (black line); black ticks indicate reflexes of the reference pattern JCPDS No. 19-0629.

In order to confirm the crystal structure of samples, crystallite and particle size, and their distribution, X-Ray powder diffraction analysis (XRD) and Transmission Electron Microscopy study (TEM) were performed. It is clear that XRD patterns of all samples (Figure S1) are in good agreement with the reference crystallographic card of Fe_3_O_4_ (PDF No. 19-0629). Peaks at 30.1, 35.4, 37.1, 43.1, 53.4, and 56.9° correspond to (220), (311), (222), (400), (422), and (511) crystallographic planes, respectively. Figure 1 shows a diffraction pattern of the MW-HS-0 sample chosen as representative. It is obvious that the samples demonstrate a high crystallinity, and there are no additional reflections, which indicates the high sample purity. In order to obtain information on lattice parameters, crystallite size and their distribution, the received XRD data were calculated by the WPPM method for all samples. This approach allows one to determine not only the volume weighted crystallite size (as the Scherrer equation does), but to calculate the weighted number.

It is necessary to note that due to similar cell parameters for magnetite (a_magnetite_ = 0.8396 nm) and maghemite (a_maghemite_ = 0.83515 nm), the difficulties in the sample phase differentiation appear. However, in our case, the lattice parameters obtained with using the WPPM method do not correspond to reference values for these oxide phases (Table 1) and are in the range of 0.8426–0.8455 nm. This phenomenon can be explained by a complex phase composition also containing another iron oxide phase (e.g., hematite) in a small amount on the nanoparticles surface (will be discussed in more detail in the Section 3.2 related to FTIR spectroscopy). Additionally, an increase in the cell parameter value is observed for the samples obtained using the humate-polyanion. Such behavior can be explained by a change in the phase composition as well as a surface relaxation model: the use of organic coating leads to nanoparticle surface relaxation from which the inflation of the interatomic distance takes place [58].

**Figure 2:**
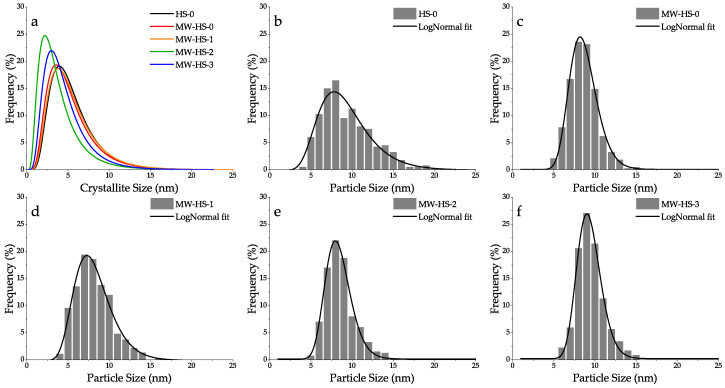
Size distribution curves of (**a**) crystallite size of all samples calculated by the WPPM method; and TEM particle size distribution curves for (**b**) HS-0, (**c**) MW-HS-0, (**d**) MW-HS-1, (**e**) MW-HS-2, (**f**) MW-HS-3 samples.

The volume weighted crystallite size seems to be similar for all the samples (Table 1), however, a slight decrease in the case of the MW-HS-3 sample is observed. In addition, WPPM offers a number of weighted crystallite size curves, which represent a view of a lognormal distribution (Figure 2a). Although all samples have the same volume weighted crystallite size, the size distribution curves have noticeable differences. The samples obtained without potassium humate (HS-0, MW-HS-0) and with post-encapsulation (MW-HS-1) show a similar mean size and standard deviation, while the samples obtained in potassium humate solutions (MW-HS-2, MW-HS-3) demonstrate a decrease in the crystallite size. This observation indicates that the polyanion participates in the crystal growth process and acts as a crystal growth suppressor, probably, due to the formation of an intermediate compound. This speculation has been confirmed by a Mössbauer study [30] where it has been shown that feroxyhyte nanoparticles formation in the presence of a humic substance proceeds via a Fe(OH)_2_ intermediate, which gives smaller nanoparticles. According to TEM images (Supplementary Figure S2), it is clear that nanoparticles of all prepared samples are of a round shape and do not contain nanoparticles of another shape, which confirms that there are no impurity phases in the samples. In Figure 2b–f, the nanoparticle size distribution curves for all samples are shown. The size distribution in all cases can be described by a lognormal function. Values of the average nanoparticles sizes and corresponding standard deviations are also summarized in Table 1. The differences in the number of weighted sizes calculated by the WPPM (Figure 2a) and obtained from TEM images (Figure 2b–f) can be explained by the fact that nanoparticles observed on TEM images are formed by both crystalline and amorphous phases, while an XRD profile gives an information on a crystallite component only. Therefore, the TEM mean size is always larger than the estimate obtained from XRD.

One can see a clear positive influence of microwave heating on the particle size: the sample obtained via a conventional thermal heating (HS-0) has a broader size distribution than that obtained in microwave conditions (MW-HS-0). The use of potassium humate and its injection time also have an impact on the nanoparticle size: in the case of the MW-HS-1 sample, humate only plays a role of a surface stabilizer due to post-encapsulation process resulting in a decreased average size and distribution, while in the case of the MW-HS-2 and MW-HS-3 samples, it is involved in both crystal growth and nanoparticles surface stabilization processes, and the obtained nanoparticles seem to be larger, because of the increased amount of the amorphous component (see Section 3.3). However, the growth of the nanoparticle size in these samples corresponds to coarsening of their crystallites.

### 3.2. Fourier Transform Infrared Spectroscopy

**Figure 3:**
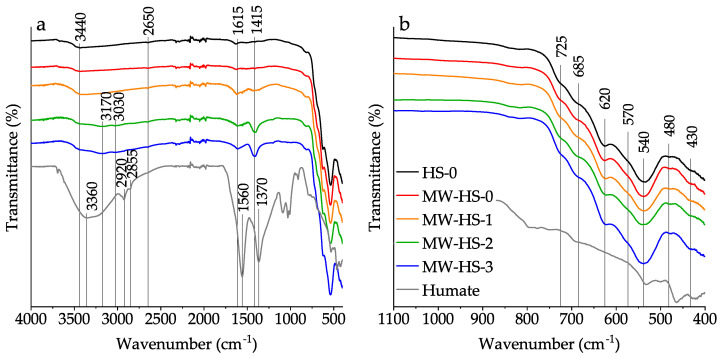
FTIR spectra (**a**) in the whole scanning range and (**b**) in the middle infrared region of the obtained samples and potassium humate powder.

All samples were studied by reflectance FTIR-spectroscopy in order to study the surface composition of magnetite nanoparticles and to confirm that the potassium humate encapsulation was successful. In Figure 3a, attenuated total reflection IR spectra of initial potassium humate and all prepared magnetite samples are shown. Upon closer examination of the middle infrared region (Figure 3b), it is getting clear that the surface composition of all magnetite-containing samples is complex. It appears that there are various iron oxide phases exhibiting Fe-O lattice vibration bands at different wavenumbers on particles surfaces [59,60,61] as follows: magnetite (a shoulder at 570 cm^−1^), maghemite (bands at 430, 620 cm^−1^ and shoulders at 685 and 725 cm^−1^), and hematite (bands at 480 and 540 cm^−1^). Also, a comparison of the recorded spectra (Figure 3a) indicates the successful polyanion adsorption on the surface of all magnetite samples. Thus, a growth of OH-groups bands is observed in the spectra of the MW-HS-1, MW-HS-2, and MW-HS-3 samples (a broad band with the maximum at 3440 cm^−1^), and the bands at 1615 and 1415 cm^−1^ corresponding to antisymmetric and symmetric stretching vibrations of carboxylate ion bonded with the magnetite surface appear. These bands correspond to the bands in the initial potassium humate spectrum with maxima at 1560 and 1370 cm^−1^. The shift of the band maxima in magnetite-containing samples is due to the formation of a weaker bond of -COO^−^ with the amphoteric iron species on the nanoparticle surface. Also, it can be seen that the magnetite samples obtained in the presence of potassium humate have different intensity ratio of the bands at 1615 and 1415 cm^−1^. In the MW-HS-2, MW-HS-3 samples, bands at 3170 and 3030 cm^−1^ corresponding to stretching vibrations of N-H and C-H bonds, respectively, appear. Based on the obtained data, we can suppose that differences in the synthetic protocol lead to differences in the chemical composition of the produced nanoparticles surface.

### 3.3. Thermal Analysis

**Figure 4:**
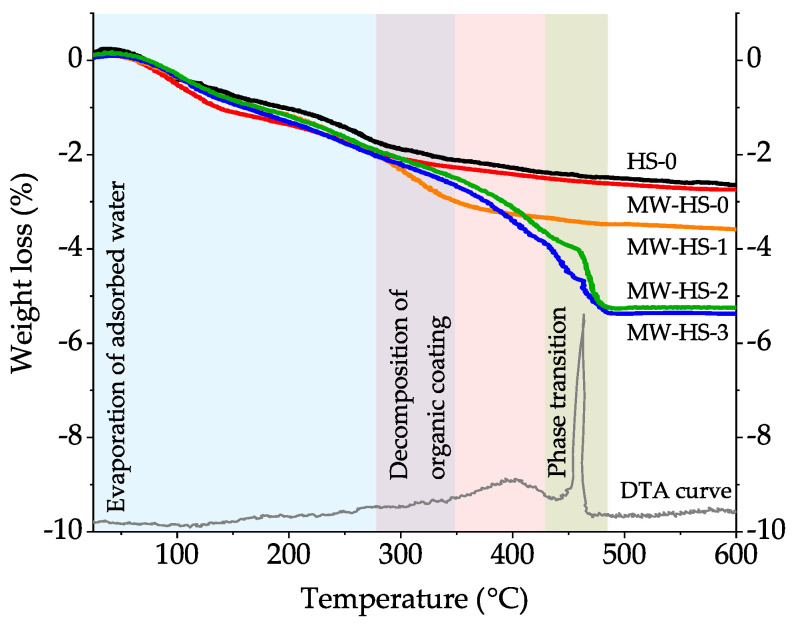
Thermogravimetric curves for the obtained samples and a representative DTA curve of the MW-HS-3 sample.

The results of the thermal analysis of the obtained samples (Figure 4) also confirm the increased amount of the amorphous phase in the samples. While comparing the samples prepared without the stabilizer (HS-0, MW-HS-0), it becomes clear that the weight loss curves behave in a similar manner: evaporation of adsorbed water on the nanoparticles surface takes place in the temperature range of up to 350 °C [62], and then the weight loss ceases gradually. There is a difference in the total weight loss (2.5% and 2.8% for HS-0 and MW-HS-0, respectively), which can be associated, probably, with the distinction in the particle size, specific surface area, and as a result, the amount of adsorbed water on the surface. In the case of MW-HS-1, the extended weight loss from 280 °C can be associated with humate decomposition on the nanoparticle surface. It seems that the coating decomposition proceeds up to exothermic phase transition followed by complete conversion of the amorphous constituent of nanoparticles, as one can see from the TGA curves of the MW-HS-2 and MW-HS-3 samples. In the temperature range of 280–450 °C, the samples MW-HS-2 and MW-HS-3 behave different from the MW-HS-1 sample and exhibit a convex curve that can be associated with differences in the composition of a coating layer. As described earlier, the curves for the samples obtained using potassium humate demonstrate a significant weight loss at the temperatures of 430–480 °C, which is due to the exothermic Fe_3_O_4_ → α-Fe_2_O_3_ phase transition (a DTA curve of the MW-HS-3 sample is chosen as a representative example) followed by the intensive decomposition of the amorphous component. As the confirmation of the phase transition, we obtained XRD patterns of the MW-HS-3 sample calcined at 400 °C and at 600 °C (Supplementary Figure S3), which confirm the crystal structure transformation of the magnetite into hematite. Also, there is a difference in the total weight loss of the potassium humate coated samples, which equals to 3.9%, 5.3%, and 5.5% for MW-HS-1, MW-HS-2, and MW-HS-3, respectively. It can be also explained by different amounts and/or composition of the adsorbed polyanion on the nanoparticles surface. Therefore, it can be concluded that the difference in the synthetic protocol influences the coating layer composition of nanoparticles, which results in differences in physical properties of the obtained nanomaterials.

### 3.4. Magnetic Measurements

The most important requirement for nanomaterials used in medicine, wastewater treatment, electronics is the magnetic susceptibility. Magnetic properties of inorganic nanomaterials depend not only on the phase composition, but also on the nanoparticle size. It is well known that when nanoparticles are reduced to a certain critical size, they become single-domain and attain superparamagnetic properties. Superparamagnetism is a specific kind of ferromagnetism occurring when nanoparticles exhibit an average zero residual magnetization in the absence of an external magnetic field. At room temperature, the critical diameter for magnetite nanoparticles is about 15–20 nm [63].

**Figure 5:**
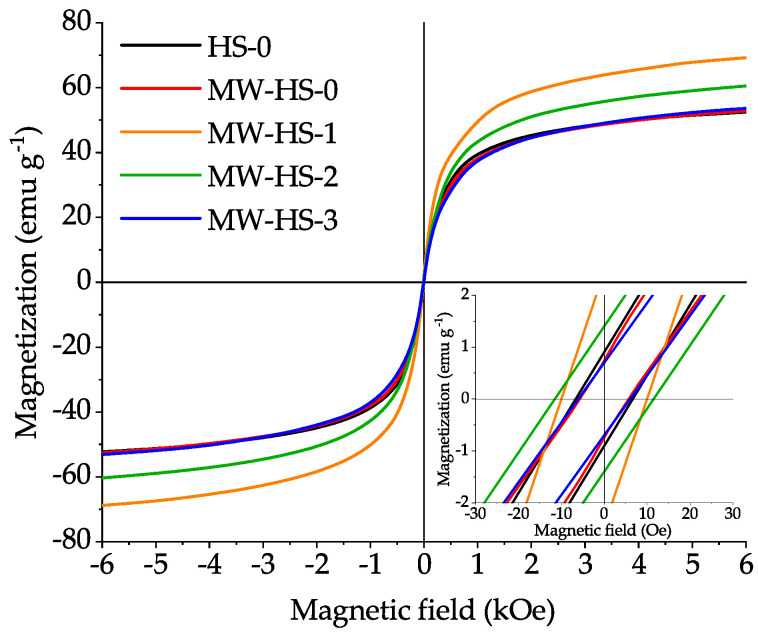
Magnetization curves of the prepared samples (inset shows a low-field region of magnetization curves to identify the coercivity and retained magnetization).

For each sample, magnetization curves were recorded at room temperature in the presence of an external magnetic field up to 6.3 kOe (Figure 5). Saturation magnetization (M_S_), which is the co-directional orientation of all magnetic moments, was calculated by the approximation of the measured magnetization values at the strong field. The saturation magnetization of the samples obtained in absence of potassium humate via conventional (HS-0) and microwave (MW-HS-0) heating has similar values, 58 and 60 emu g^−1^, respectively (Table 2). However, the samples obtained in the presence of the stabilizer show different saturation magnetization values: 80, 68, and 60 emu g^−1^ for MW-HS-1, MW-HS-2, and MW-HS-3, respectively. This behavior cannot be explained by the size effect, because nanoparticles of all coated samples are in the same size range. The size distributions are also quite narrow. Moreover, the MW-HS-0 sample has the narrowest distribution. However, this does not demonstrate high magnetization, which is similar to the uncoated sample value. It is reasonable to assume that the key factor influencing the magnetic properties is the polyanion adsorbed on the nanoparticle surface. There are studies describing the magnetic properties of magnetite coated with humate, their results show that in comparison with unstabilized nanoparticles, samples with a stabilizer have a lower magnetization value in the range of 50–60 emu g^−1^ [64,65]. In these works, the weakening of magnetic properties is associated with a decrease in the mass fraction of the magnetic component in the composite. We have found the opposite effect as a result of the synthesis of the MW-HS-1, MW-HS-2 samples. Thereby, when using the natural polyanion as a stabilizer, a noticeable increase in the magnetization of the sample is observed. When comparing the results of our study and of the previously described studies, we can conclude that such a difference in M_S_ with similar average sizes of nanoparticles is observed due to the difference in mass fractions of the organic compound adsorbed on the surface of the nanoparticles. Thus, in the above studies, the mass fraction of the natural polyanion reaches 10%, which leads to a noticeable decrease in the magnetization per unit mass. However, the MW-HS-1 sample, which represents magnetite nanoparticles with a mass fraction of organic stabilizer not exceeding 1 wt.% (according to TGA measurements), shows a noticeable increase in saturation magnetization, which corresponds to 80 emu g^–1^. This unusual behavior is primarily due to the interaction of the functional groups of the polyanion with iron atoms on the surface of the nanoparticles. The most probable assumption is the interaction of the carboxy group with surface iron atoms, as was shown earlier when using another capping agent, including oleic acid [66,67,68,69]. It is surprising that, in order to achieve improved magnetic and colloidal (will be considered later) properties, it is not necessary to stabilize the entire surface of nanoparticles, but it is sufficient to stabilize only the most active metal atoms to achieve a Fe-O bond length on the surface of the nanoparticle comparable with the bond length in the bulk [70]. This hypothesis is confirmed by the fact that with an increase in the mass fraction of adsorbed polyanion on the surface of nanoparticles, a noticeable decrease in their magnetic properties is observed (samples MW-HS-2, MW-HS-3 with a content of organic component on the surface of nanoparticles of 2.5 and 2.6 wt.%, respectively). Thus, we can say that the selection of the optimal amount of a stabilizer and the capping method of the surface of magnetic materials, in particular magnetite, plays a tremendous role in its magnetic properties.

All samples show an extremally low coercive field (below 15 Oe) and magnetic remanence (smaller than 2 emu g^−1^), which indicates their superparamagnetic character.

### 3.5. Colloid Properties

The use of nanoparticles in the field of medicine imposes additional limitations on the properties of nanoparticles associated primarily with properties of their surfaces. Uncoated magnetite nanoparticles show a high stability in solutions with high and low pH values [71]. However, their use for in vivo application necessitates surface stabilization for a number of reasons: enhancing of biocompatibility, protection of the magnetic core from oxidation, reduction of interaction with nonspecific cells, enhancement of colloidal stability in the region of neutral solutions, and changing of surface charge [12,72]. The ζ-potential is considered to be a key factor influencing the colloid stability. Colloid solutions with a high ζ-potential are electrically stabilized, while low ζ-potential solutions are inclined to coagulate and flocculate.

**Figure 6:**
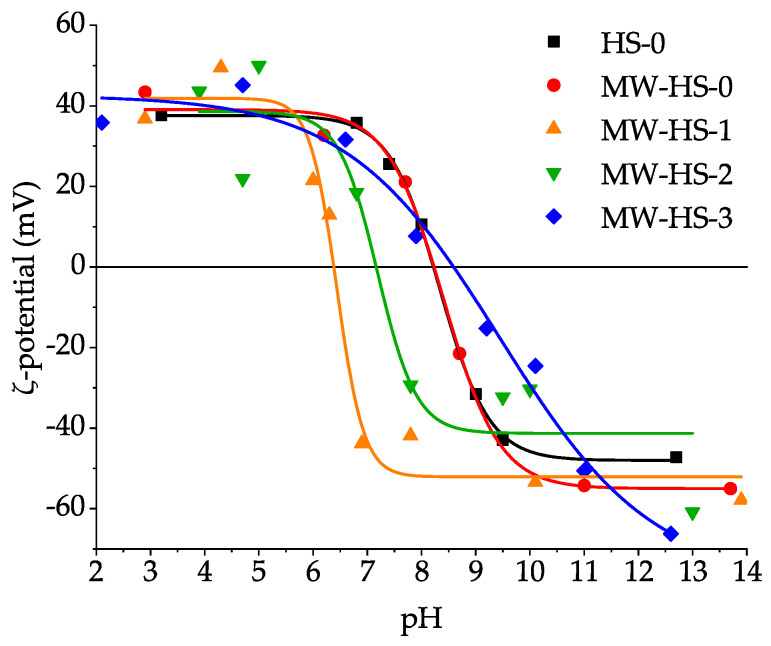
ζ-potentials of all samples in water solutions in a wide pH range.

Figure 6 shows the ζ-potentials of all samples in water solutions in a wide pH range. It is clearly seen that the samples obtained without the stabilizer (HS-0 and MW-HS-0) have almost the same potential behavior and the point of zero charge (PZC) at pH 8.3, which confirms the same surface composition (Table 2). This fact correlates well with the results of IR spectroscopy and TG study. The obtained pH value of PZC is slightly different from the value (pH 7.9 ± 0.1) measured by another research group [45]. Quite interesting results are obtained for humate coated magnetite nanoparticles. The post-encapsulation (MW-HS-1 sample) results in a PZC shift to a pH of 6.4. Moreover, an increase in the inflection of the potential curve is observed, which leads to a high stability of the colloid (ζ = −43 mV) even at pH 6.9. The change in the sequence of potassium humate injection to the reaction mixture also affects the stability of nanoparticles in solution. Thus, in the MW-HS-2 and MW-HS-3 samples, the PZC shifts toward high pH values of 7.2 and 9.4, respectively. Also, the change in the nature of the inflection of the potential curve takes place. By comparing the results with thermogravimetric data, one can assume that the amount of adsorbed polyanion leads to a change in the properties of the colloid. Also, one of the reasons for the PZC shift of the colloid solutions of MW-HS-2 and MW-HS-3 samples may be a change in the phase composition of the surface oxide layer of the nanoparticles, namely, the formation of a larger amount of hematite, which has a PZC at higher pH values than magnetite [73]. This is confirmed by a change in the unit cell parameters from 0.8429 to 0.8455 nm for the MW-HS-1 and MW-HS-3 samples, respectively (see Section 3.1), as well as by the results of IR-spectroscopy (see Section 3.2). Thus, it can be concluded that, for medical purposes, particularly for in vivo administration, the post-encapsulation is more favorable because of the high stability of the colloid at pH values typical for blood (pH ~7.3–7.4).

Supplementary Figure S4 presents the distribution curves of the hydrodynamic size of nanoparticles; the mean size and deviation for all the samples are given in Table 2. The highest hydrodynamic size of 130.7 ± 36.7 nm is found for the MW-HS-1 sample. The samples obtained with the stabilizer have significantly smaller sizes of 41.5 ± 13.5 and 34.9 ± 11.5 nm for the MW-HS-2 and MW-HS-3 samples, respectively. Such a behavior can be explained by the fact that during nanoparticles dispersion, freshy distilled water is acidified by CO_2_ from the air to pH of 6.5. At this pH, the stability of the MW-HS-1 colloid is very low, which results in an agglomeration of nanoparticles [74]. The samples MW-HS-2 and MW-HS-3 have PZC at higher pH, resulting in a decrease in the hydrodynamic diameter. It is not difficult to see that nanoparticles obtained without encapsulation show a similar hydrodynamic size with the MW-HS-3 sample, and their values are equal to 35.7 ± 12.0 and 52.3 ± 17.2 nm for the HS-0 and MW-HS-0 samples, respectively.

**Figure 7:**
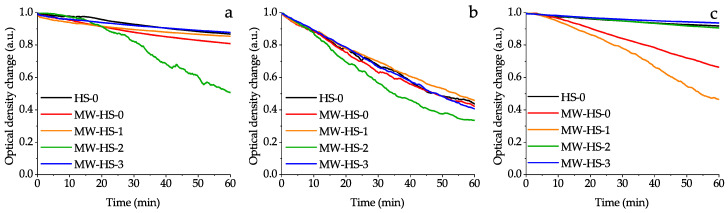
Optical density changes at the wavelength 600 nm for the iron oxide colloids in: (**a**) deionized water; (**b**) normal saline solution; (**c**) model infusion solution.

In order to evaluate colloidal stability of the synthesized magnetic nanoparticles, the stability tests in water, normal saline and model infusion solutions were performed (Figure 7). It is evident that the medium has an influence on the colloidal stability of magnetic iron oxide nanoparticles. In general, the behavior of the synthesized nanoparticles corresponds to conventional trends. Thus, nanoparticles, dispersed in water demonstrate a moderate stability (excluding the MW-HS-2 sample) and complete sedimentation occurs for 24 h. Samples dispersed in the normal saline show a much lower stability due to increased ionic strength and they coagulate several hours afterwards. However, in the case of the model infusion solution, which consisted of NaCl, albumin, and glucose, most of the samples (HS-0, MW-HS-2, and MW-HS-3) have high colloidal stability, at least by seven days. In combination with excellent magnetic properties, these samples are excellent candidates for biomedical applications.

### 3.6. Summary

It can be concluded that the use of microwave irradiation as the heating source in the preparation process has an impact on the size of nanoparticles, which can be associated with a more uniform heating of the entire mixture and completeness of the crystallization of an amorphous phase. Samples obtained without the stabilizer at conventional thermal heating and microwave conditions have the same weighted crystallite size but differ from each other as TEM measurements show. On the other hand, properties of the samples obtained by both methods do not differ significantly as indicated by the results of other analyses, in particular, thermogravimetric analysis, which can be explained by the same ratios of crystalline and amorphous phases in the samples.

Also, it was shown that potassium humate used as a stabilizer has significant impact on the structural and physical properties of the obtained nanoparticles. In particular, it leads to a decrease of the crystallite size, while at the same time, increasing particle size and a narrower particle size distribution are observed. It appears that pre-synthesis dissolution of potassium humate seems to change the reaction mechanism, which proceeds through the formation of an intermediate, resulting in the decreased crystallite size and increased monodispersity of the obtained samples. Surface stabilization results in the enhanced stability of magnetite nanoparticles in water solutions at blood pH values with the point of zero charge of 6.4. The value of PZC can be shifted up to pH 9.4 by variation in the synthetic protocol. Additionally, changing the stabilization protocol allows one to obtain highly stable nanoparticles (samples MW-HS-2 and MW-HS-3) in model infusion solutions (for at least one week), which can be used in medical purposes. Moreover, it was also shown that the humate polyanion has an impact on the magnetic properties of the magnetite nanoparticles: a variation in the synthetic protocol changes the saturation magnetization of the samples from 60 to 80 emu g^−1^. So, the post-synthesis capped magnetite sample (MW-HS-1) shows the highest saturation magnetization value (80 emu g^−1^), which is close to the bulk magnetite. However, the MW-HS-3 sample obtained from a solution with preliminarily dissolved potassium humate demonstrates a magnetization value which is similar to that for uncapped samples.

## 4. Conclusions

Thus, a series of monodisperse magnetite nanoparticles coated with a hydrophilic humate polyanion was successfully synthesized by a facile microwave-assisted method without using an inert atmosphere. Additionally, we synthesized two samples without coating by both conventional heating and a microwave-assisted approach. It was shown that microwave heating results in more uniform nanoparticles. Also, we established that the coating protocol significantly changes the properties of nanopowders. The obtained samples are superparamagnetic with enhanced saturation magnetization and are highly stable in model infusion solutions, which makes them good candidates for biomedical in-vivo applications, for instance, for drug delivery purposes, hyperthermia, etc. The obtained findings are helpful for materials scientists who are in search of choosing an appropriate strategy and synthesis conditions of hydrophilic magnetic nanoparticles with specific physical properties.

## Figures and Tables

**Table 1:** Synthesis conditions and structural properties of the samples.

Sample	Synthesis Method	Use of Potassium Humate	Volume Weighted Crystallite Size, WPPM (nm)	Lattice Parameter (nm)	Average Particle Size, TEM (nm)
HS-0	thermal	not used	8.3	0.8426	9.3 ± 3.3
MW-HS-0	microwave	not used	8.6	0.8429	8.7 ± 1.7
MW-HS-1	microwave	after precipitation	8.7	0.8429	8.2 ± 2.3
MW-HS-2	microwave	before precipitation	8.8	0.8431	8.4 ± 1.6
MW-HS-3	microwave	before salts dissolution	7.9	0.8455	9.4 ± 1.5

**Table 2:** Magnetic and colloidal properties of the obtained samples.

Sample	Saturation Magnetization (emu g^−1^)	Point of Zero Charge (pH)	Average Hydrodynamic Diameter (nm)
HS-0	58	8.33 ± 0.04	35.7 ± 12.0
MW-HS-0	60	8.41 ± 0.17	52.3 ± 17.2
MW-HS-1	80	6.44 ± 0.11	130.7 ± 36.7
MW-HS-2	68	7.18 ± 0.36	41.5 ± 13.5
MW-HS-3	60	9.44 ± 0.44	34.9 ± 11.5

## Supplementary Materials

The following are available online at https://www.mdpi.com/2079-4991/10/8/1558/s1, Figure S1: XRD patterns of the samples (gray dots) with WPPM fitting (black line); black ticks indicate reflexes of the reference pattern JCPDS No. 19-0629. Figure S2: TEM images of prepared samples: (**a**) HS 0, (**b**) MW HS 0, (**c**) MW HS 1, (**d**) MW HS 2, (**e**) MW HS 3. Figure S3: XRD patterns of the MW-HS-3 sample calcined at 400 °C corresponding to a magnetite phase (PDF No. 19-0629) and at 600 °C corresponding to a hematite phase (PDF No. 33-0664). Figure S4: Distribution curves of hydrodynamic diameter of obtained samples in water solutions.

## References

[1] Su C. (2017). Environmental implications and applications of engineered nanoscale magnetite and its hybrid nanocomposites: A review of recent literature. J. Hazard. Mater..

[2] Tang S.C.N., Lo I.M.C. (2013). Magnetic nanoparticles: Essential factors for sustainable environmental applications. Water Res..

[3] Tursunov O., Kustov L., Kustov A.A. (2017). Brief Review of Carbon Dioxide Hydrogenation to Methanol Over Copper and Iron Based Catalysts. Oil Gas Sci. Technol.–Revue d’IFP Energies nouvelles.

[4] Dobrowolski J.W., Bedla D., Czech T., Gambuś F., Górecka K., Kiszczak W., Kuźniar T., Mazur R., Nowak A., Śliwka M. (2017). Integrated Innovative Biotechnology for Optimization of Environmental Bioprocesses and a Green Economy. Optimization and Applicability of Bioprocesses.

[5] Sharma R.K., Dutta S., Sharma S., Zboril R., Varma R.S., Gawande M.B. (2016). Fe_3_O_4_ (iron oxide)-supported nanocatalysts: Synthesis, characterization and applications in coupling reactions. Green Chem..

[6] Tarasov A.L., Kostyukhin E.M., Kustov L.M. (2018). Gasification of metal-containing coals and carbons via their reaction with carbon dioxide. Mendeleev Commun..

[7] Skomski R. (2003). Nanomagnetics. J. Phys. Condens. Matter.

[8] De B., Yadav A., Khan S., Kar K.K. (2017). A Facile Methodology for the Development of a Printable and Flexible All-Solid-State Rechargeable Battery. ACS Appl. Mater. Interfaces.

[9] Bruck A.M., Cama C.A., Gannett C.N., Marschilok A.C., Takeuchi E.S., Takeuchi K.J. (2016). Nanocrystalline iron oxide based electroactive materials in lithium ion batteries: The critical role of crystallite size, morphology, and electrode heterostructure on battery relevant electrochemistry. Inorg. Chem. Front..

[10] Revia R.A., Zhang M. (2016). Magnetite nanoparticles for cancer diagnosis, treatment, and treatment monitoring: Recent advances. Mater. Today.

[11] Javed Y., Akhtar K., Anwar H., Jamil Y. (2017). MRI based on iron oxide nanoparticles contrast agents: Effect of oxidation state and architecture. J. Nanopart. Res..

[12] Hu Y., Mignani S., Majoral J.-P., Shen M., Shi X. (2018). Construction of iron oxide nanoparticle-based hybrid platforms for tumor imaging and therapy. Chem. Soc. Rev..

[13] Wu K., Su D., Liu J., Saha R., Wang J.-P. (2019). Magnetic nanoparticles in nanomedicine: A review of recent advances. Nanotechnology.

[14] Katz E. (2020). Magnetic Nanoparticles. Magnetochemistry.

[15] Majidi S., Zeinali Sehrig F., Farkhani S.M., Soleymani Goloujeh M., Akbarzadeh A. (2016). Current methods for synthesis of magnetic nanoparticles. Artif. Cells Nanomed. Biotechnol..

[16] Duan M., Shapter J.G., Qi W., Yang S., Gao G. (2018). Recent progress in magnetic nanoparticles: Synthesis, properties, and applications. Nanotechnology.

[17] Katz E. (2019). Synthesis, Properties and Applications of Magnetic Nanoparticles and Nanowires—A Brief Introduction. Magnetochemistry.

[18] Baghbanzadeh M., Carbone L., Cozzoli P.D., Kappe C.O. (2011). Microwave-Assisted Synthesis of Colloidal Inorganic Nanocrystals. Angew. Chem. Int. Ed..

[19] Kostyukhin E.M., Kustov L.M. (2018). Microwave-assisted synthesis of magnetite nanoparticles possessing superior magnetic properties. Mendeleev Commun..

[20] Vikanova K., Redina E., Kapustin G., Nissenbaum V., Mishin I., Kostyukhin E., Kustov L. (2020). Template-free one-step synthesis of micro-mesoporous CeO_2_–ZrO_2_ mixed oxides with a high surface area for selective hydrogenation. Ceram. Int..

[21] Kostyukhin E.M., Kustov A.L., Kustov L.M. (2019). One-step hydrothermal microwave-assisted synthesis of LaFeO_3_ nanoparticles. Ceram. Int..

[22] Gawande M.B., Shelke S.N., Zboril R., Varma R.S. (2014). Microwave-Assisted Chemistry: Synthetic Applications for Rapid Assembly of Nanomaterials and Organics. Acc. Chem. Res..

[23] Mallakpour S., Madani M.A. (2015). Review of current coupling agents for modification of metal oxide nanoparticles. Prog. Org. Coat..

[24] De Melo B.A.G., Motta F.L., Santana M.H.A. (2016). Humic acids: Structural properties and multiple functionalities for novel technological developments. Mater. Sci. Eng. C.

[25] Tang Z., Zhao X., Zhao T., Wang H., Wang P., Wu F., Giesy J.P. (2016). Magnetic Nanoparticles Interaction with Humic Acid: In the Presence of Surfactants. J. Environ. Sci. Technol..

[26] Polyakov A.Y., Lebedev V.A., Shirshin E.A., Rumyantsev A.M., Volikov A.B., Zherebker A., Garshev A.V., Goodilin E.A., Perminova I.V. (2017). Non-classical growth of water-redispersible spheroidal gold nanoparticles assisted by leonardite humate. CrystEngComm.

[27] Jung B., O’Carroll D., Sleep B. (2014). The influence of humic acid and clay content on the transport of polymer-coated iron nanoparticles through sand. Sci. Total Environ..

[28] Polyakov A.Y., Goldt A.E., Sorkina T.A., Perminova I.V., Pankratov D.A., Goodilin E.A., Tretyakov Y.D. (2012). Constrained growth of anisotropic magnetic δ-FeOOH nanoparticles in the presence of humic substances. CrystEngComm.

[29] Chekanova A.E., Sorkina T.A., Dubov A.L., Nikiforov V.N., Davydova G.A., Selezneva I.I., Goodilin E.A., Trusov L.A., Korolev V.V., Aref’ev I.M. (2009). New environmental nontoxic agents for the preparation of core-shell magnetic nanoparticles. Mendeleev Commun..

[30] Polyakov A.Y., Sorkina T.A., Goldt A.E., Pankratov D.A., Perminova I.V., Goodilin E.A. (2013). Mössbauer spectroscopy of frozen solutions as a stepwise control tool in preparation of biocompatible humic-stabilized feroxyhyte nanoparticles. Hyperfine Interact..

[31] Li Y., Yang C., Guo X., Dang Z., Li X., Zhang Q. (2015). Effects of humic acids on the aggregation and sorption of nano-TiO_2_. Chemosphere.

[32] Mert E.H., Yıldırım H., Üzümcü A.T., Kavas H. (2013). Synthesis and characterization of magnetic polyHIPEs with humic acid surface modified magnetic iron oxide nanoparticles. React. Funct. Polym..

[33] Liu J., Zhao Z., Jiang G. (2008). Coating Fe3O4 Magnetic Nanoparticles with Humic Acid for High Efficient Removal of Heavy Metals in Water. Environ. Sci. Technol..

[34] Jiang W., Cai Q., Xu W., Yang M., Cai Y., Dionysiou D.D., O’Shea K.E. (2014). Cr(VI) Adsorption and Reduction by Humic Acid Coated on Magnetite. Environ. Sci. Technol..

[35] Niu H., Zhang D., Zhang S., Zhang X., Meng Z., Cai Y. (2011). Humic acid coated Fe3O4 magnetic nanoparticles as highly efficient Fenton-like catalyst for complete mineralization of sulfathiazole. J. Hazard. Mater..

[36] Zhang X., Zhang P., Wu Z., Zhang L., Zeng G., Zhou C. (2013). Adsorption of methylene blue onto humic acid-coated Fe_3_O_4_ nanoparticles. Colloids Surf. A.

[37] Schepetkin I., Khlebnikov A., Kwon B.S. (2002). Medical drugs from humus matter: Focus on mumie. Drug Dev. Res..

[38] Van Rensburg C.E.J. (2015). The Antiinflammatory Properties of Humic Substances: A Mini Review. Phytother. Res..

[39] Jansen van Rensburg C.E., Naude P.J. (2009). Potassium Humate Inhibits Complement Activation and the Production of Inflammatory Cytokines In Vitro. Inflammation.

[40] Illés E., Tombácz E. (2006). The effect of humic acid adsorption on pH-dependent surface charging and aggregation of magnetite nanoparticles. J. Colloid Interface Sci..

[41] Tombácz E., Tóth I.Y., Nesztor D., Illés E., Hajdú A., Szekeres M., Vékás L. (2013). Adsorption of organic acids on magnetite nanoparticles, pH-dependent colloidal stability and salt tolerance. Colloids Surf. A.

[42] Chekli L., Phuntsho S., Tijing L.D., Zhou J.L., Kim J.H., Shon H.K. (2014). Stability of Fe-oxide nanoparticles coated with natural organic matter under relevant environmental conditions. Water Sci. Technol..

[43] Hajdú A., Illés E., Tombácz E., Borbáth I. (2009). Surface charging, polyanionic coating and colloid stability of magnetite nanoparticles. Colloids Surf. A.

[44] Govan J., Gun’ko Y. (2014). Recent Advances in the Application of Magnetic Nanoparticles as a Support for Homogeneous Catalysts. Nanomaterials.

[45] Illés E., Tombácz E. (2003). The role of variable surface charge and surface complexation in the adsorption of humic acid on magnetite. Colloids Surf. A.

[46] Jolivet J.-P., Chanéac C., Tronc E. (2004). Iron oxide chemistry. From molecular clusters to extended solid networks. Chem. Commun..

[47] Saxena N., Singh M. (2017). Efficient synthesis of superparamagnetic magnetite nanoparticles under air for biomedical applications. J. Magn. Magn. Mater..

[48] Scardi P., Ortolani M., Leoni M. (2010). WPPM: Microstructural Analysis beyond the Rietveld Method. Mater. Sci. Forum.

[49] Stingaciu M., Andersen H.L., Granados-Miralles C., Mamakhel A., Christensen M. (2017). Magnetism in CoFe_2_ O_4_ nanoparticles produced at sub- and near-supercritical conditions of water. CrystEngComm.

[50] Leoni M., Confente T., Scardi P. (2006). PM2K: A flexible program implementing Whole Powder Pattern Modelling. Z. Kristallogr. Suppl.

[51] Kachala V.V., Khemchyan L.L., Kashin A.S., Orlov N.V., Grachev A.A., Zalesskiy S.S., Ananikov V.P. (2013). Target-oriented analysis of gaseous, liquid and solid chemical systems by mass spectrometry, nuclear magnetic resonance spectroscopy and electron microscopy. Russ. Chem. Rev..

[52] Kirichenko O.A., Kapustin G.I., Tkachenko O.P., Nissenbaum V.D., Mishin I.V., Davshan N.A., Redina E.A., Kustov L.M. (2016). Synthesis and characterization of novel Au/θ-Al_2-x_Fe_x_O_3_ nanomaterials with high thermal stability in catalytic oxidation of carbon monoxide. Mater. Res. Bull..

[53] Chernavskii P.A., Pankina G.V., Lunin V.V. (2011). Magnetometric methods of investigation of supported catalysts. Russ. Chem. Rev..

[54] Abramenko N.B., Demidova T.B., Abkhalimov E.V., Ershov B.G., Krysanov E.Y., Kustov L.M. (2018). Ecotoxicity of different-shaped silver nanoparticles: Case of zebrafish embryos. J. Hazard. Mater..

[55] Bhattacharya S., Mallik D., Nayar S. (2011). Comparative Study of Biomimetic Iron Oxides Synthesized Using Microwave Induced and Conventional Method. IEEE Trans. Magn..

[56] Obaidat I., Issa B., Haik Y. (2015). Magnetic Properties of Magnetic Nanoparticles for Efficient Hyperthermia. Nanomaterials.

[57] Hedayatnasab Z., Abnisa F., Daud W.M.A.W. (2017). Review on magnetic nanoparticles for magnetic nanofluid hyperthermia application. Mater. Des..

[58] Sciancalepore C., Bondioli F., Manfredini T., Gualtieri A. (2015). Quantitative phase analysis and microstructure characterization of magnetite nanocrystals obtained by microwave assisted non-hydrolytic sol–gel synthesis. Mater. Charact..

[59] Namduri H., Nasrazadani S. (2008). Quantitative analysis of iron oxides using Fourier transform infrared spectrophotometry. Corros. Sci..

[60] Belin T., Guigue-Millot N., Caillot T., Aymes D., Niepce J. (2002). Influence of Grain Size, Oxygen Stoichiometry, and Synthesis Conditions on the γ-Fe_2_O_3_ Vacancies Ordering and Lattice Parameters. J. Solid State Chem..

[61] Hu L., Percheron A., Chaumont D., Brachais C.-H. (2011). Microwave-assisted one-step hydrothermal synthesis of pure iron oxide nanoparticles: Magnetite, maghemite and hematite. J. Sol-Gel Sci. Technol..

[62] Chen Y.H. (2013). Thermal properties of nanocrystalline goethite, magnetite, and maghemite. J. Alloys Compd..

[63] Alp E., Aydogan N. (2016). A comparative study: Synthesis of superparamagnetic iron oxide nanoparticles in air and N_2_ atmosphere. Colloids Surf. A.

[64] Aparicio F., Escalada J.P., De Gerónimo E., Aparicio V.C., García Einschlag F.S., Magnacca G., Carlos L., Mártire D.O. (2019). Carbamazepine Degradation Mediated by Light in the Presence of Humic Substances-Coated Magnetite Nanoparticles. Nanomaterials.

[65] Lu M., Zhang Y., Zhou Y., Su Z., Liu B., Li G., Jiang T. (2019). Adsorption-desorption characteristics and mechanisms of Pb(II) on natural vanadium, titanium-bearing magnetite-humic acid magnetic adsorbent. Powder Technol..

[66] Cîrcu M., Nan A., Borodi G., Liebscher J., Turcu R. (2016). Refinement of Magnetite Nanoparticles by Coating with Organic Stabilizers. Nanomaterials.

[67] Kandasamy G., Surendran S., Chakrabarty A., Kale S.N., Maity D. (2016). Facile synthesis of novel hydrophilic and carboxyl-amine functionalized superparamagnetic iron oxide nanoparticles for biomedical applications. RSC Adv..

[68] Patsula V., Kosinová L., Lovrić M., Ferhatovic Hamzić L., Rabyk M., Konefal R., Paruzel A., Šlouf M., Herynek V., Gajović S. (2016). Superparamagnetic Fe_3_O_4_ Nanoparticles: Synthesis by Thermal Decomposition of Iron(III) Glucuronate and Application in Magnetic Resonance Imaging. ACS Appl. Mater. Interfaces.

[69] Guardia P., Labarta A., Batlle X. (2011). Tuning the Size, the Shape, and the Magnetic Properties of Iron Oxide Nanoparticles. J. Phys. Chem. C.

[70] Salafranca J., Gazquez J., Pérez N., Labarta A., Pantelides S.T., Pennycook S.J., Batlle X., Varela M. (2012). Surfactant Organic Molecules Restore Magnetism in Metal-Oxide Nanoparticle Surfaces. Nano Lett..

[71] Veiseh O., Gunn J.W., Zhang M. (2010). Design and fabrication of magnetic nanoparticles for targeted drug delivery and imaging. Adv. Drug Deliv. Rev..

[72] Mohammed L., Gomaa H.G., Ragab D., Zhu J. (2017). Magnetic nanoparticles for environmental and biomedical applications: A review. Particuology.

[73] Qin X., Liu F., Wang G., Huang G. (2015). Adsorption of humic acid from aqueous solution by hematite: Effects of pH and ionic strength. Environ. Earth Sci..

[74] Vega-Chacón J., Arbeláez M.I.A., Jorge J.H., Marques R.F.C., Jafelicci M. (2017). pH-responsive poly(aspartic acid) hydrogel-coated magnetite nanoparticles for biomedical applications. Mater. Sci. Eng. C.

