# Supplementary material for: Microwave-Assisted Synthesis of Water-Dispersible Humate-Coated Magnetite Nanoparticles: Relation of Coating Process Parameters to the Properties of Nanoparticles
Source: Nanomaterials (Basel). 2020 Aug 8;10(8):1558. doi: 10.3390/nano10081558 (PMC7466618; doi:10.3390/nano10081558)
Supplement: Supplementary file 1 [file nanomaterials-10-01558-s001.pdf]

nanomaterials

Article

Microwave-Assisted Synthesis of Water-Dispersible Humate-Coated Magnetite Nanoparticles: Relation of Coating Process Parameters to the Properties of Nanoparticles

Egor M. Kostyukhin [1,2,*], Vera D. Nissenbaum [1], Evgeny V. Abkhalimov [3], Alexander L. Kustov [1,2,4], Boris G. Ershov [3] and Leonid M. Kustov [1,2,4,*]

1. Laboratory of Development and Study of Polyfunctional Catalysts, N.D. Zelinsky Institute of Organic Chemistry RAS, 47 Leninsky prosp., Moscow 119991 Russia; vdn14@inbox.ru (V.D.N.); kyst@list.ru (A.L.K.)
2. Laboratory of Nanochemistry and Ecology, National University of Science and Technology MISiS, 4 Leninsky prosp., Moscow 119049, Russia
3. Laboratory of Radiation-Induced Chemical Transformations of Materials, A.N. Frumkin Institute of Physical Chemistry and Electrochemistry RAS, 31 Leninsky Prospect, bldg. 4, Moscow 119071, Russia; abkhalimov@ipc.rssi.ru (E.V.A.); ershov@ipc.rssi.ru (B.G.E.)
4. Laboratory of Ecological Chemistry, Chemistry Department, M.V. Lomonosov Moscow State University, 1 Leninskie Gory, bldg. 3, Moscow 119991, Russia

* Correspondence: kostyuhin.egor@gmail.com (E.M.K.); lmkustov@mail.ru (L.M.K.)

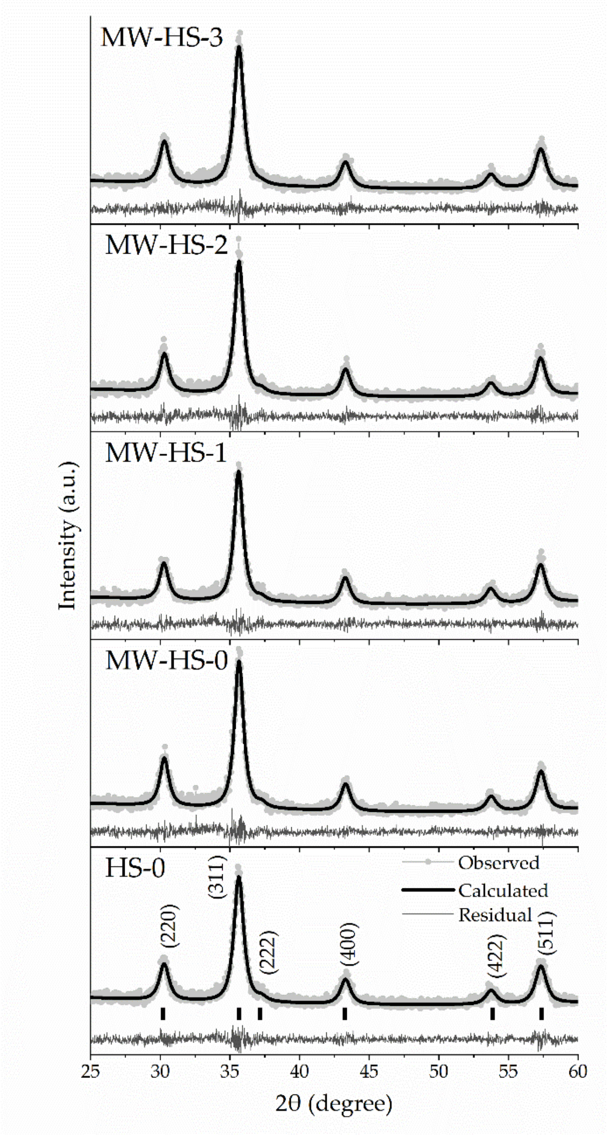

Figure S1. XRD patterns of the samples (gray dots) with WPPM fitting (black line); black ticks indicate reflexes of the reference pattern JCPDS No. 19-0629.

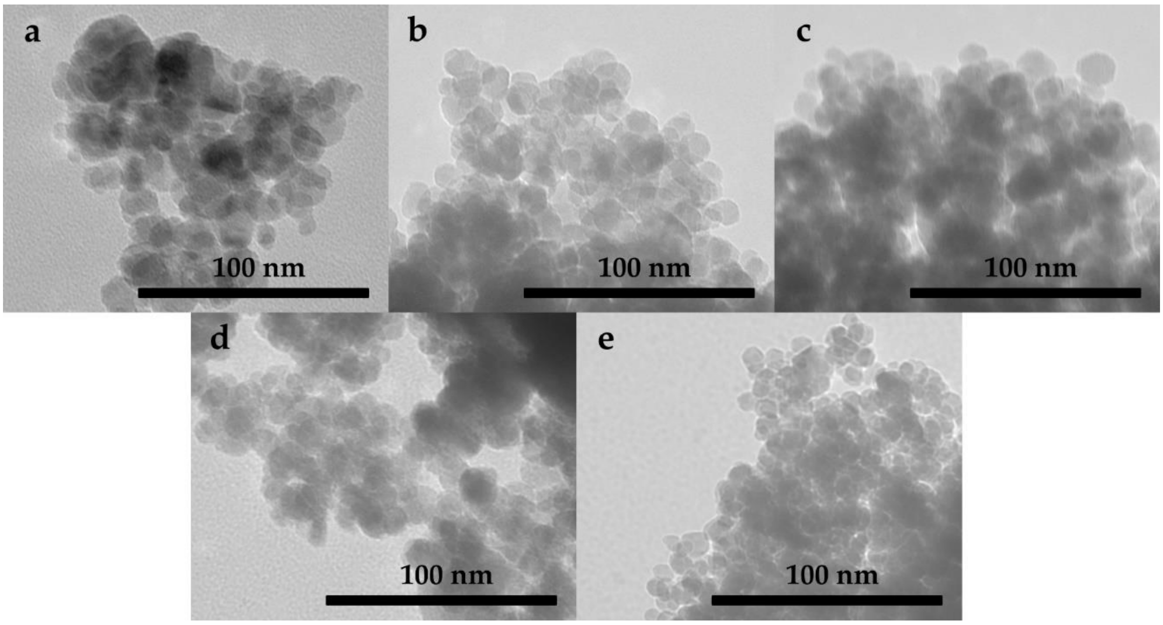

Figure S2. TEM images of prepared samples: (**a**) HS-0, (**b**) MW-HS-0, (**c**) MW-HS-1, (**d**) MW-HS-2, (**e**) MW-HS-3.

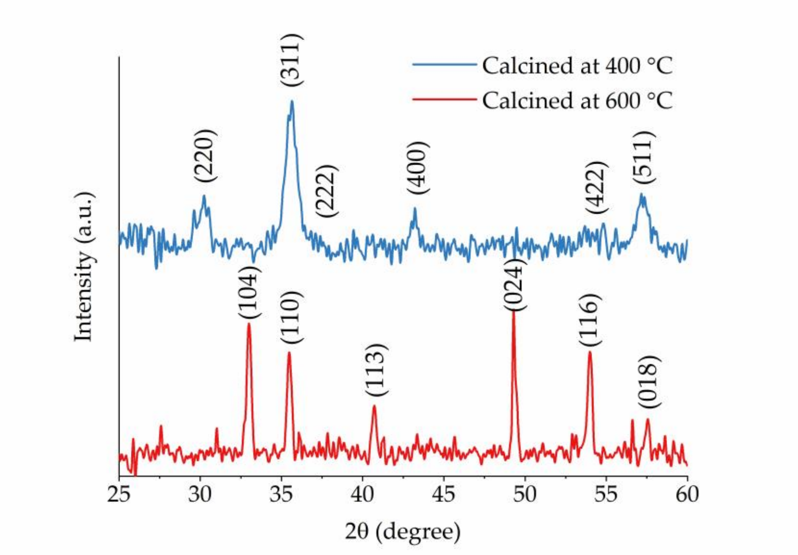

Figure S3. XRD patterns of the MW-HS-3 sample calcined at 400 °C corresponding to a magnetite phase (PDF No. 19-0629) and at 600 °C corresponding to a hematite phase (PDF No. 33-0664).

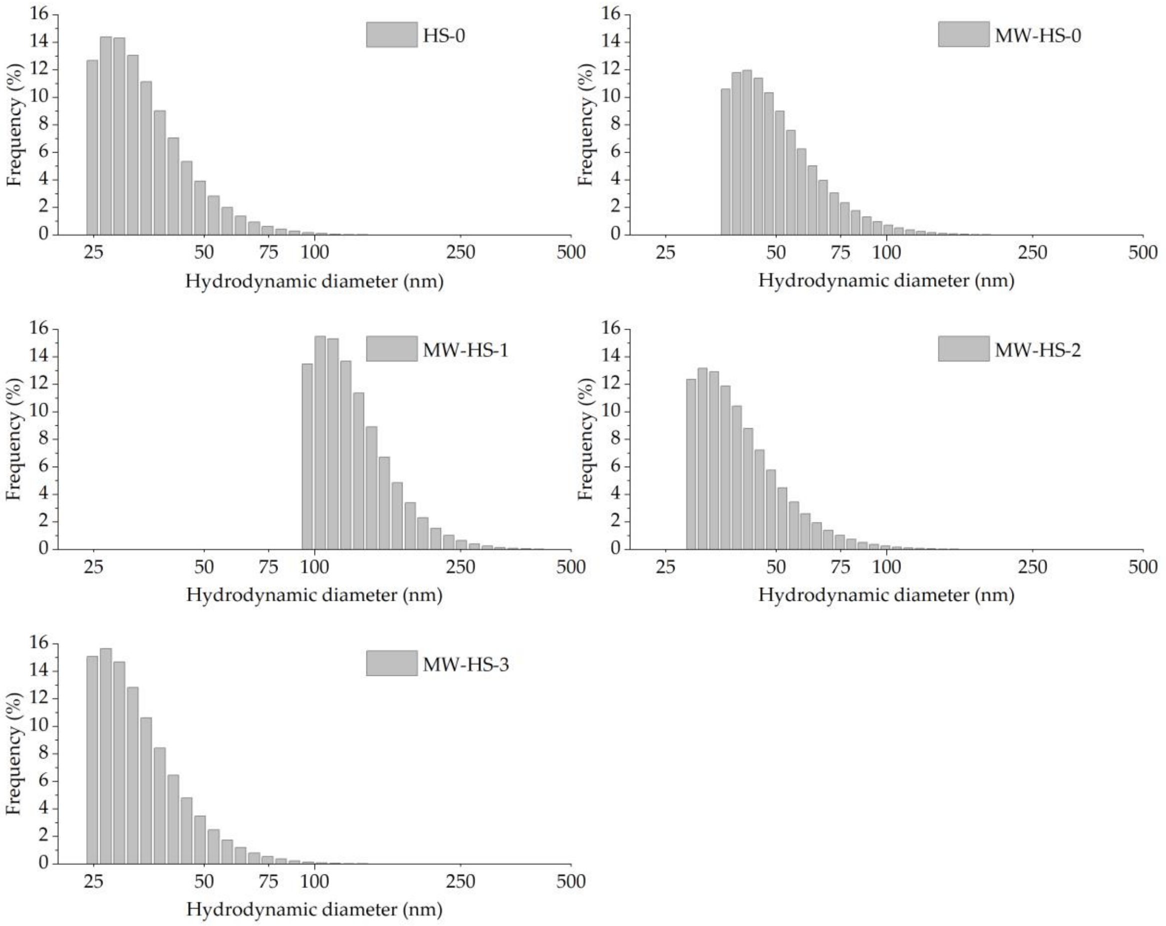

Figure S4. Distribution curves of hydrodynamic diameter of obtained samples in water solutions.

© 2020 by the authors. Licensee MDPI, Basel, Switzerland. This article is an open access article distributed under the terms and conditions of the Creative Commons Attribution (CC BY) license (http://creativecommons.org/licenses/by/4.0/).
